# Temporal Dynamics of Perioperative Redox Balance and Its Association with Postoperative Delirium After Cardiac Surgery

**DOI:** 10.3390/antiox15010108

**Published:** 2026-01-14

**Authors:** Yukiko Arai, Yoshihisa Koyama, Ayako Takahashi, Shoichi Shimada, Takeshi Yoshida

**Affiliations:** 1 Department of Anesthesiology and Intensive Care Medicine, Graduate School of Medicine, The University of Osaka, Suita 565-0871, Japan; u768564h@ecs.osaka-u.ac.jp (Y.A.); atakahashi@anes.med.osaka-u.ac.jp (A.T.); takeshiyoshida@hp-icu.med.osaka-u.ac.jp (T.Y.); 2 Department of Neuroscience and Cell Biology, Graduate School of Medicine, The University of Osaka, Suita 565-0871, Japan; shimada@anat1.med.osaka-u.ac.jp; 3 Addiction Research Unit, Osaka Psychiatric Research Center, Osaka Psychiatric Medical Center, Osaka 541-8567, Japan

**Keywords:** postoperative delirium, oxidative stress, redox balance, cardiac surgery, derivatives of reactive oxygen metabolites, biological antioxidant potential

## Abstract

Postoperative delirium (POD) is a neurocognitive complication that commonly occurs after cardiac surgery. Despite the association of POD with increased morbidity and mortality, reliable perioperative biomarkers for predicting POD remain scarce. This retrospective observational study investigated whether temporal changes in perioperative redox balance are associated with POD development. Fifty adult patients who underwent elective cardiac surgery at Osaka University Hospital were included. Serum levels of derivatives of reactive oxygen metabolites (d-ROMs) and biological antioxidant potential (BAP) were measured preoperatively, immediately after intensive care unit admission, and on postoperative days 1–4. POD was assessed twice daily using the Intensive Care Delirium Screening Checklist (ICDSC), with an ICDSC score of ≥3 indicating delirium. POD occurred in 18 (36%) out of 50 patients. Compared with non-POD patients, those with POD exhibited higher preoperative d-ROMs levels, a lower BAP/d-ROMs ratio, a transient postoperative increase in BAP, and a relatively higher BAP/d-ROMs ratio during the early postoperative period. Preoperative d-ROMs levels showed a positive correlation with the maximum ICDSC score. In conclusion, perioperative redox dynamics are associated with POD risk and severity. Redox-related markers, particularly d-ROMs, may have potential as biomarkers for identifying patients at higher risk of POD after cardiac surgery, and their clinical utility warrants further prospective validation.

## 1. Introduction

Postoperative delirium (POD) is an acute neurocognitive complication that commonly occurs following anesthesia and surgery [1]. A subset of patients has been reported to experience persistent cognitive decline or to progress to dementia [2]. POD is considered a major patient safety concern owing to its association with prolonged hospitalization, delayed rehabilitation, reduced quality of life, increased burden on the healthcare system, and increased mortality [3]. POD is also increasingly recognized as an important indicator of healthcare quality in geriatric medicine [4,5,6]. Nevertheless, diagnosing POD poses a challenge because symptoms fluctuate throughout the day and may be masked by sedatives [7]. Moreover, the underlying mechanisms of POD remain poorly understood, and no prophylactic or therapeutic pharmacological interventions have yet been established, with management relying primarily on nonpharmacological strategies [8].

Although the incidence of POD varies depending on surgery type, it is particularly high at 25–52% after cardiovascular surgery [9,10]. Furthermore, several cases remain unrecognized, with up to 85% of potentially undiagnosed cases [3,11]. Cardiovascular surgery induces postoperative oxidative stress due to systemic and local alterations in perfusion and oxygenation, cardiopulmonary bypass, and abrupt fluctuations in plasma pH and cellular metabolism [12,13,14,15,16]. This oxidative injury has been suggested to contribute to POD development; however, the specific mechanisms remain unclear, and reliable biomarkers for predicting or diagnosing POD have not yet been identified. Various oxidative stress biomarkers, including lipid peroxidation products, 4-hydroxynonenal, malondialdehyde, isoprostanes, 8-hydroxy-2′-deoxyguanosine, thymine glycol, and protein carbonyls, have been investigated [17]. Each biomarker reflects distinct oxidative pathways, and reactive oxygen species have an extremely short half-life; consequently, measuring and interpreting these biomarkers are challenging in clinical settings.

Recently, derivatives of reactive oxygen metabolites (d-ROMs) and biological antioxidant potential (BAP) have attracted attention as simple indicators for the evaluation of oxidative stress dynamics [18,19,20,21,22,23,24]. d-ROMs measure circulating organic hydroperoxides, thereby reflecting the total oxidative products derived from ROS [18], whereas BAP captures the integrated antioxidant capacity of plasma constituents such as uric acid, thiol compounds, and vitamins [16]. d-ROMs and BAP assays can be performed more rapidly than conventional oxidative stress markers using standard automated biochemical analyzers and are suitable for point-of-care testing, supporting their potential application in routine clinical practice. Furthermore, the BAP/d-ROMs ratio is considered an integrated indicator that reflects the relative balance between oxidative stress and antioxidant capacity. While d-ROMs alone represent oxidative burden and BAP alone reflects antioxidant capacity, their ratio provides a more comprehensive assessment of how effectively the body compensates for oxidative stress or, conversely, whether antioxidant defenses are impaired [25]. Previous studies have reported that increased oxidative stress, neuroinflammation, mitochondrial dysfunction, and disruption of the blood–brain barrier contribute to the development of POD [2,12,13,17]. Therefore, evaluating not only d-ROMs and BAP individually but also the BAP/d-ROMs ratio as an indicator of perioperative redox balance may offer clinically meaningful insights into the pathophysiology of POD. However, the clinical utility of d-ROMs, BAP, and BAP/d-ROMs ratio for predicting and diagnosing POD has not been sufficiently investigated. In particular, studies exploring its perioperative temporal dynamics during the early postoperative period are limited. Therefore, we aimed to address this knowledge gap by retrospectively observing daily changes in d-ROMs and BAP levels from the preoperative period to postoperative day 4, and by examining their associations (rather than causal effects) with the development of POD.

This exploratory study was designed to clarify whether temporal redox fluctuations relate to the timing of POD onset, and to assess the potential utility of these biomarkers as perioperative monitoring indicators.

## 2. Methods

### 2.1. Study Design

This retrospective observational study included adult patients aged ≥18 years who underwent cardiac surgery (valvular surgery, aortic surgery, or coronary artery bypass grafting [CABG]) from August 2024 to June 2025 at Osaka University Hospital (Suita, Osaka, Japan). Patients who underwent heart transplantation or ventricular assist device implantation were excluded. Residual serum specimens from routine perioperative clinical laboratory testing were retrospectively used to measure the d-ROMs and BAP levels. No additional blood sampling for research purposes was performed.

This study was conducted in accordance with the principles embodied in the Declaration of Helsinki and was approved by the Ethics Committee of Osaka University Hospital (approval No. 23190; approval date: 17 October 2023). Informed consent was obtained from all subjects involved in the study.

### 2.2. Patients

During the study period, 175 patients met the inclusion criteria. Following the exclusion of patients who underwent emergency surgery, were diagnosed with dementia preoperatively, developed postoperative stroke, required re-sternotomy or re-intubation, and did not have sufficient residual serum samples for biomarker measurement, 50 patients were ultimately included in the analysis.

### 2.3. Blood Analysis

Oxidative stress markers were measured using a REDOXLIBRA analyzer (WISMERLL Co., Ltd., Tokyo, Japan). Circulating hydroperoxides were quantified as an index of oxidative stress using the d-ROMs test, and blood antioxidant capacity was assessed based on colorimetric reduction reactions using the BAP test. Reference ranges for healthy adults are provided in Table S1. The BAP/d-ROMs ratio derived from these measurements served as an integrated index of systemic redox balance by reflecting both oxidative stress and antioxidant capacity. Thus, the BAP/d-ROMs ratio was included as an outcome measure in this study.

**Figure 1:**
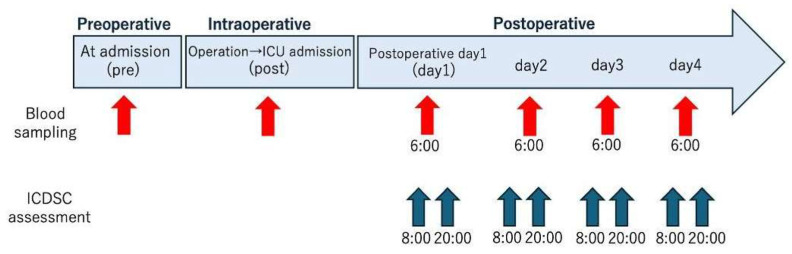
Timeline of perioperative blood sampling and postoperative delirium (POD) assessment. The red arrows indicate the time points at which blood samples were collected: at hospital admission (labeled as “pre”), immediately after surgery at ICU admission (labeled as “post”), and at 6:00 on postoperative days 1–4 (labeled as “days 1–4”). The blue arrows indicate the time points of Intensive Care Delirium Screening Checklist (ICDSC) assessment, which was performed twice daily at 08:00 and 20:00 from day 1 to day 4.

Blood samples were obtained at six time points: preoperatively (baseline), after surgery at intensive care unit (ICU) admission, and on postoperative days 1–4 (Figure 1). The follow-up period was set to postoperative day 4 because POD is most frequently observed within 1–3 days after surgery [26]. Residual serum specimens from routine perioperative blood testing were used in all measurements.

### 2.4. POD Assessment

Trained ICU nurses assessed POD twice daily using the Intensive Care Delirium Screening Checklist (ICDSC), beginning on postoperative day 1. The ICDSC is an 8-item tool that assesses acute changes in consciousness, inattention, disorientation, and other delirium symptoms, with scores ranging from 0 to 8. Evaluations were conducted only when the Richmond Agitation–Sedation Scale (RASS) score ranged from −2 to +4; assessments were deferred when the RASS score was ≤−4. POD was defined using an ICDSC score of ≥3 based on a previous study that recommended a score of ≥3 as the optimal cutoff for clinical delirium diagnosis [27].

### 2.5. Study Outcomes

The primary outcome was the association between POD and perioperative changes in oxidative stress markers (d-ROMs, BAP, and BAP/d-ROMs ratio) from baseline to postoperative day 4. The secondary outcome was the relationship between preoperative oxidative stress status and POD severity, as assessed by Pearson’s correlation between preoperative biomarker values and the highest ICDSC score recorded during the observation period.

### 2.6. Statistical Analysis

Continuous variables are presented as means ± standard deviations. Associations between POD and longitudinal trajectories of oxidative stress markers were analyzed using a mixed-effects model for repeated measures (MMRM). Fixed effects included the time point (categorical), group (POD vs. non-POD), and their interaction (time × group). A compound symmetry covariance structure was applied for within-patient covariance. Least-squares (LS) means and standard errors (SEs) were estimated, and *p*-values for the overall difference between POD and non-POD groups and the interaction term were calculated. A covariate-adjusted model that included age, hypertension, and operative time was additionally used (the rationale behind covariate selection is described in the “Results” section). Between-group differences at each time point were examined using contrast analyses within the same MMRM (exploratory, without multiplicity adjustment). Associations between preoperative biomarker levels and the maximum ICDSC score were explored through Pearson’s correlation analysis after confirming bivariate normality using standard bivariate diagnostic ellipses. All statistical tests were two-sided, with the significance level set at *p* < 0.05. Statistical analyses were performed using JMP Student Edition 18 (SAS Institute Inc., Cary, NC, USA).

## 3. Results

### 3.1. Patient Characteristics

POD occurred in 18 (36%) out of 50 patients included in this study. The baseline characteristics of patients are presented in Table 1. Patients with POD were significantly older than those without POD (75.6 ± 6.7 years vs. 69.3 ± 11.6 years, *p* = 0.019). No significant differences in sex, body mass index, smoking history, and alcohol consumption were observed. Among oxidative stress markers, preoperative d-ROMs levels showed a trend toward higher values in the POD group.

Hypertension was more prevalent in the POD group (72.2% vs. 37.5%); other comorbidities did not differ significantly. With respect to the operative factors, the operative time was significantly longer in the POD group (373 ± 153 min vs. 260 ± 74 min, *p* < 0.001). Total blood product transfusion, anesthesia time, cardiopulmonary bypass time, and the proportion of aortic surgery also tended to be higher in the POD group; however, these parameters are highly interrelated and considered to reflect surgical invasiveness. Given the limited sample size of this study, including multiple correlated invasiveness variables simultaneously in the model could lead to overfitting; therefore, operative time was selected as a representative operative factor in subsequent analyses. The surgical approach (minimally invasive cardiac surgery vs. median sternotomy) did not differ significantly between the groups.

For covariate determination, factors known to affect both oxidative stress markers and POD were evaluated based on the above findings and previous studies. Age, hypertension, and operative time were chosen as covariates in the adjustment analyses based on previous studies reporting that age was a major risk factor for POD [2,28] and was closely associated with increased oxidative stress [29]; that operative time was linked to both increased surgical stress and cardiopulmonary bypass exposure, potentially affecting POD [30] and oxidative stress [31]; and that hypertension contributed to chronic oxidative stress [32] and may impair cerebrovascular autoregulation [33]. Additionally, patients with cerebrovascular disease carried an increased POD risk [30], implicating hypertension as a possible mediator of vulnerability.

### 3.2. Temporal Trajectories and Time-Point Comparisons of Oxidative Stress Markers

**Figure 2:**
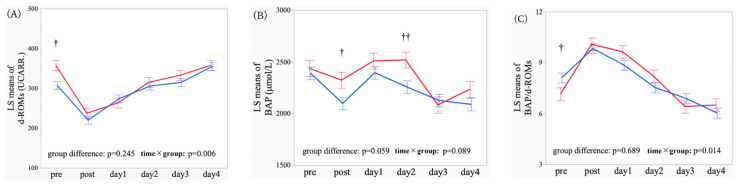
Longitudinal profiles of derivatives of reactive oxygen metabolites (d-ROMs) (**A**) biological antioxidant potential (BAP) (**B**) and BAP/d-ROMs ratio (**C**) in patients with and without postoperative delirium (POD). At each time point, values are least-squares (LS) means estimates adjusted by a mixed-effects model for repeated measures (MMRM). Lines connect the LS means across time, and error bars indicate the SEs. The time points were as follows: pre = preoperative baseline; post (day 0) = immediately after surgery at ICU admission; days 1–4 = postoperative days 1–4. Red indicates POD (+), whereas blue denotes POD (−). The *p*-values shown in each panel correspond to tests for the group differences (POD vs. non-POD) and the time × group interaction (fixed effects) in the MMRM. †: *p* < 0.05, ††: *p* < 0.01 for between-group comparisons at each time point.

Longitudinal trajectories of d-ROMs, BAP, and BAP/d-ROMs ratio from the preoperative period to postoperative day 4 were compared between patients with and without POD. For d-ROMs, there was no significant overall difference between POD and non-POD groups (*p* = 0.245); in contrast, the time × group interaction was significant (*p* = 0.006), indicating differing d-ROMs temporal trajectories between the two groups despite similar overall mean values (Figure 2A). For BAP, both the overall group difference (*p* = 0.059) and interaction (*p* = 0.089) showed a trend toward significance, with the POD group attaining higher overall BAP values and exhibiting a distinct temporal pattern (Figure 2B). For the BAP/d-ROMs ratio, the overall group difference was not significant (*p* = 0.689), whereas the interaction was significant (*p* = 0.014), suggesting divergent BAP/d-ROMs trajectories (Figure 2C).

Because multiple comparisons were performed, the following time-point findings should be interpreted as exploratory. Between-group differences at individual time points were further evaluated through exploratory contrast analyses within the same MMRM. Preoperative d-ROMs levels were significantly higher in the POD group (*p* = 0.002). BAP was significantly higher immediately after ICU admission and on postoperative day 2 (*p* = 0.022 and *p* = 0.008), whereas the preoperative BAP/d-ROMs ratio was significantly lower (*p* = 0.046) (Figure S1).

**Figure 3:**
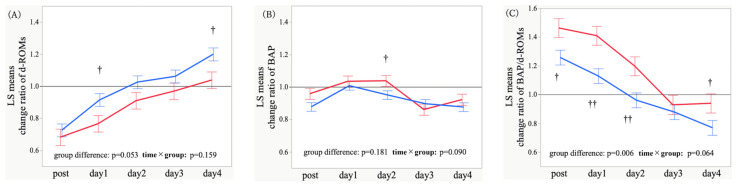
Longitudinal profiles of the change ratio (value at each time point divided by the preoperative baseline value, which was set to 1) for derivatives of reactive oxygen metabolites (d-ROMs) (**A**), biological antioxidant potential (BAP) (**B**), and BAP/d-ROMs ratio (**C**) in patients with and without postoperative delirium (POD). At each point, values are least-squares (LS) means estimates adjusted by a mixed-effects model for repeated measures (MMRM) Lines connect the LS means across time, and error bars indicate the SEs. The time points were as follows: pre = preoperative baseline; post (day 0) = immediately after surgery at ICU admission; days 1–4 = postoperative days 1–4. Red indicates POD (+), whereas blue denotes POD (−). The *p*-values shown in each panel correspond to tests for the overall group difference (POD vs. non-POD) and the time × group interaction (fixed effects) in the MMRM. †: *p* < 0.05, ††: *p* < 0.01 for between-group differences at each time point.

Relative changes from baseline (expressed as the ratio to preoperative levels) were also analyzed to account for interindividual variability. Regarding relative changes in d-ROMs levels, the overall group difference showed a borderline significance (*p* = 0.053), and the time × group interaction was not significant (*p* = 0.159), indicating lower overall postoperative d-ROMs increases but similar temporal trajectories in the POD group (Figure 3A). With respect to relative changes in BAP, the group difference was not significant (*p* = 0.181); however, the interaction exhibited a trend toward significance (*p* = 0.090), suggesting a possible trend toward differential postoperative BAP dynamics (Figure 3B). As for relative changes in the BAP/d-ROMs ratio, both the group difference (*p* = 0.006) and interaction (*p* = 0.064) were significant or showed a trend toward significance, indicating a consistently higher BAP/d-ROMs ratio and a distinct trajectory in the POD group (Figure 3C). Exploratory contrasts revealed a significantly smaller relative increase in d-ROMs levels on postoperative days 1 and 4 (*p* = 0.025 and *p* = 0.016), a significantly greater relative change in BAP on postoperative day 2 (*p* = 0.044), and a significantly higher relative increase in the BAP/d-ROMs ratio from immediately after ICU admission to postoperative days 2 and 4 (*p* = 0.015, *p* = 0.001, *p* = 0.005, and *p* = 0.046) (Figure S2). These contrasts should be viewed as hypothesis-generating rather than confirmatory.

### 3.3. Adjusted Temporal Trajectories and Time-Point Comparisons of Oxidative Stress Markers

**Figure 4:**
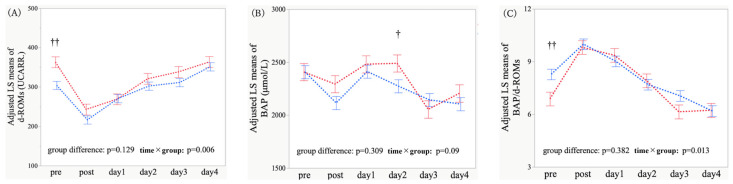
Longitudinal profiles of derivatives of reactive oxygen metabolites (d-ROMs) (**A**), biological antioxidant potential (BAP) (**B**), and BAP/d-ROMs ratio (**C**) in patients with and without postoperative delirium (POD) after adjustment for potential confounders. At each time point, values are least-squares (LS) mean estimates adjusted by a mixed-effects model for repeated measures (MMRM), with hypertension status, age, and operative time included as covariates. Lines connect the LS means across time, and error bars indicate the SEs. The time points were as follows: pre = preoperative baseline; post (day 0) = immediately after surgery at ICU admission; days 1–4 = postoperative days 1–4. Red indicates POD (+), whereas blue denotes POD (−). The *p*-values shown in each panel correspond to tests for the main effect of POD and the time × POD interaction (fixed effects) in the covariate-adjusted MMRM. †: *p* < 0.05, ††: *p* < 0.01 for between-group comparisons at each time point.

Age, hypertension, and operative time were included as covariates in the MMRM to control for potential confounding. After adjustment, the overall group difference in d-ROMs between POD and non-POD groups was not significant (*p* = 0.129); conversely, the time × group interaction was significant (*p* = 0.006), suggesting distinct temporal d-ROMs trajectories (Figure 4A). As for BAP, neither the group difference nor the interaction was significant (*p* = 0.309 and *p* = 0.090) (Figure 4B). Regarding the BAP/d-ROMs ratio, the group difference remained non-significant (*p* = 0.382), whereas the interaction was significant (*p* = 0.013), indicating differing postoperative BAP/d-ROMs dynamics (Figure 4C). Exploratory contrasts revealed higher preoperative d-ROMs levels in the POD group (*p* = 0.001) and higher BAP on postoperative day 2 (*p* = 0.045) (Figure S3). Overall, the primary findings were largely preserved after adjustment.

**Figure 5:**
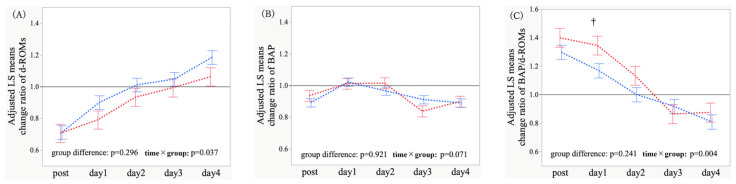
Longitudinal profiles of the change ratio (value at each time point divided by the preoperative baseline value, which was set to 1) for derivatives of reactive oxygen metabolites (d-ROMs) (**A**), biological antioxidant potential (BAP) (**B**), and BAP/d-ROMs ratio (**C**) in patients with and without postoperative delirium (POD) after adjustment for potential confounders. At each time point, values are least-squares (LS) mean estimates adjusted by an mixed-effects model for repeated measures (MMRM), with age, hypertension status, and operative time included as covariates. Lines connect the LS means across time, and error bars indicate the SEs. The time points were as follows: pre = preoperative baseline; post (day 0) = immediately after surgery at ICU admission; days 1–4 = postoperative days 1–4. Red indicates POD (+), whereas blue denotes POD (−). The *p*-values shown in each panel correspond to tests for the overall group difference (POD vs. non-POD) and the time × group interaction (fixed effects) in the covariate-adjusted MMRM. †: *p* < 0.05, for between-group comparisons at each time point.

Covariate-adjusted analysis of relative changes revealed that the overall group difference on relative changes in d-ROMs was not significant (*p* = 0.296); however, the time × group interaction was significant (*p* = 0.037), implying continued divergence in postoperative d-ROMs trajectories (Figure 5A). Regarding relative changes in BAP, neither the group difference nor the interaction was significant (*p* = 0.921 and *p* = 0.071) (Figure 5B). As for relative changes in the BAP/d-ROMs ratio, the overall group difference was not significant (*p* = 0.241), whereas the interaction was significant (*p* = 0.004), suggesting differential restoration of oxidative balance (Figure 5C). Time-point contrasts revealed an attenuated relative increase in d-ROMs on postoperative days 1 and 4 (*p* = 0.090 and *p* = 0.060) and a greater relative increase in the BAP/d-ROMs ratio on postoperative days 1 and 2 (*p* = 0.015 and *p* = 0.059) (Figure S4). Several between-group differences were attenuated by adjustment; nevertheless, the overall directionality and distinct oxidative stress responses associated with POD were maintained. Given the number of exploratory contrasts, these results should be interpreted cautiously and considered hypothesis-generating.

### 3.4. Association Between Oxidative Stress Markers and ICDSC Scores

**Figure 6:**
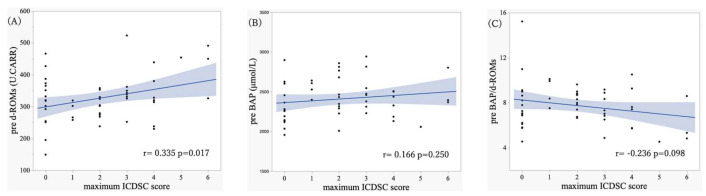
Correlations between preoperative oxidative stress markers and the maximum Intensive Care Delirium Screening Checklist (ICDSC) score. Scatter plots illustrate the correlations between the maximum ICDSC score and preoperative derivatives of reactive oxygen metabolites (d-ROMs) levels (**A**), preoperative biological antioxidant potential (BAP) (**B**), and preoperative BAP/d-ROMs ratio (**C**). The shaded areas indicate the 95% confidence intervals around the regression lines.

As a secondary analysis, the association between preoperative oxidative stress markers (d-ROMs, BAP, and BAP/d-ROMs ratio) and the maximum ICDSC score was explored. On Pearson’s correlation analysis, preoperative d-ROMs levels showed a significant positive correlation with the maximum ICDSC score (*r* = 0.335, *p* = 0.017) (Figure 6A). In contrast, preoperative BAP values were not significantly correlated with the maximum ICDSC score (*r* = 0.166, *p* = 0.250) (Figure 6B), nor was the preoperative BAP/d-ROMs ratio (*r* = −0.236, *p* = 0.098) (Figure 6C). These findings indicated that higher preoperative oxidative stress levels may be associated with increased POD severity.

## 4. Discussion

This study investigated the association between POD and oxidative stress markers (namely, d-ROMs, BAP, and BAP/d-ROMs ratio) in patients who underwent cardiac surgery. Patients with POD exhibited significantly higher d-ROMs levels preoperatively (Figure 4A), lower BAP/d-ROMs ratio at baseline (Figure 4C), transiently elevated BAP values postoperatively, and relatively higher BAP/d-ROMs ratio during the early postoperative phase (Figure 4B and Figure 5C). Preoperative d-ROMs levels showed a significant positive correlation with the maximum ICDSC score (Figure 6A), suggesting that a greater preoperative oxidative stress burden may contribute to increased POD severity.

### 4.1. Comparison with Previous Studies

Previous studies have highlighted the contribution of intraoperative or postoperative oxidative damage and impaired antioxidant defenses to POD development. For instance, López et al. reported an association between oxidative injury during cardiac surgery and the onset of POD [13], and Iizuka et al. reported that postoperative decline in plasma ascorbic acid levels was linked to POD risk [34]. Other studies suggest that preoperative disturbances in redox balance may predispose patients to POD. Kaźmierski et al. demonstrated that reduced preoperative plasma antioxidant capacity was associated with POD and negatively correlated with the postoperative levels of soluble receptor for advanced glycation end products [35].

Consistent with these findings, our results indicated elevated preoperative d-ROMs levels and reduced BAP/d-ROMs ratio in the POD group, suggesting that vulnerability to surgical stress may be reflected in a dysregulated redox profile before surgery. Additionally, the POD group had transiently elevated BAP values postoperatively and a relatively higher BAP/d-ROMs ratio early after surgery, indicating a potentially exaggerated reactive antioxidant response. Although such a response may appear protective, one possible interpretation is that a heightened antioxidant activation could be conceptually consistent with the notion of “reductive stress,” a state characterized by excessive antioxidant activity and redox shift toward reduction; however, this remains speculative. Reductive stress has been reported to induce pathological cardiac remodeling and impair diastolic function, and these alterations are attenuated by glutathione depletion [36]. Reductive stress has also been identified as a potential early marker of Alzheimer’s disease [37], suggesting a link to neurocognitive impairment.

Given that POD involves functional alterations in cognition-related brain regions such as the hippocampus and frontal cortex [38,39], such postoperative antioxidant activation observed in our cohort could represent one possible pathophysiological response associated with POD. However, this should not be interpreted as definitive evidence of mechanistic involvement. Notably, the timing of POD onset in our patients, primarily on postoperative days 1–2, aligned closely with the peak temporal changes in these markers. Collectively, these findings expand upon previous reports by underscoring the importance of both preexisting redox vulnerability and early postoperative dynamic responses. However, alternative interpretations are possible, and because intracellular reductive state markers such as Reduced glutathione/oxidized glutathione and Nicotinamide adenine dinucleotide phosphate (reduced form)/nicotinamide adenine dinucleotide phosphate (oxidized form) were not directly assessed in the present study, the relationship between postoperative antioxidant elevation and reductive stress should be regarded as hypothesis-generating requiring further validation.

In our study, the time × group interaction was marginally significant. This noteworthy finding suggests that the trajectories of perioperative redox dynamics may differ between the two groups. Redox homeostasis is maintained not only by quantitative shifts in oxidant–antioxidant balance but also by a complex regulatory signaling network [40], and disruption of redox resilience has been implicated in pathological processes [41]. The temporal dynamics of redox responses have been proposed to be clinically relevant in the perioperative setting [42], and static measurements conducted at a single time point may not sufficiently capture their biological significance.

Temporal changes in BAP and BAP/d-ROMs ratio showed greater fluctuations with more wave-like patterns in the POD group, whereas a more linear and stable progression was observed in the non-POD group. This divergence was not large and did not reach statistical significance in the exploratory analyses. Nevertheless, this finding raises the possibility that, in addition to quantitative differences, temporal instability of redox regulatory capacity (redox instability) contributes to vulnerability to POD. Therefore, although conventional quantitative markers remain fundamental in POD research, incorporating an additional perspective that considers the temporal regulation of redox homeostasis may provide further insights into the underlying pathophysiology.

### 4.2. Secondary and Subgroup Analyses

In the secondary analysis, preoperative d-ROMs levels showed a small-to-moderate positive correlation with the maximum ICDSC score, indicating that greater baseline oxidative stress may contribute to POD risk and severity. Previous studies on POD severity have largely focused on biomarkers related to neuronal injury, such as S100B and NfL [43]; nonetheless, evidence linking oxidative stress markers to POD severity remains limited. Thus, our findings provide new insights; however, the correlation strength was modest and does not imply causality, thereby warranting further investigation.

Subgroup analyses stratified by age, operative time, and hypertension status were also performed to explore potential effect modification (Figures S5–S7). Due to the limited sample size in each subgroup, covariate adjustment was not performed, and analyses were conducted exploratorily using raw measurements only; therefore, statistical power and result stability may be limited. Age ≥ 75 years was selected to reflect demographic characteristics and Japanese clinical standards [44,45], and the median operative time (268 min) was used as an objective cutoff. Results for patients aged <75 years closely resembled the main findings, whereas no significant effects in patients aged ≥75 years were observed (Figure S5A–C). Similarly, significant temporal differences emerged only in the surgery subgroup with a longer operative time (Figure S6A–C). Finally, results were consistent with the main analysis only in patients with hypertension (Figure S7A–C).

Taken together, these results indicate that redox alterations associated with POD may be less apparent in older adults, whereas patients undergoing highly invasive procedures or those with chronic oxidative stress backgrounds, such as hypertension, may be more vulnerable to surgery-induced oxidative stress, potentially exhibiting more pronounced redox abnormalities. However, given the exploratory nature of the analysis, this interpretation should be regarded as hypothesis-generating.

### 4.3. Clinical Implications

Our findings suggest that redox dynamics from the preoperative period to the early postoperative period may be relevant to POD prediction and severity stratification. The presence of increased preoperative d-ROMs levels in the POD group and its correlation with the maximum ICDSC score suggest that preoperative oxidative stress assessment may help in identifying high-risk patients. In fact, when compared with the reference ranges (Supplementary Table S1), the covariate-adjusted preoperative d-ROMs level in the POD group corresponded to the middle-level oxidative stress category (363.2 ± 13.7 U.CARR), whereas the non-POD group fell within the borderline-level range (304.2 ± 10.4 U.CARR), indicating that the two groups differed by approximately one stress category. This categorical difference may also have clinical implications.

In addition, the transient postoperative increase in BAP and BAP/d-ROMs ratio observed in the early postoperative period may reflect dynamic redox fluctuations driven by a transient activation of antioxidant responses, and this period also corresponded to the time when POD was most frequently observed. Specifically, on postoperative day 2, BAP values remained within the normal reference range in both groups (2489.4 ± 80.9 μmol/L in the POD group vs. 2273.9 ± 61.8 μmol/L in the non-POD group; Supplementary Table S1), yet the POD group showed an increase of approximately 200 μmol/L (around 10%) from baseline. This magnitude was comparable to the width of the reference range, suggesting that a more pronounced antioxidant response may have been elicited in the POD group even within the normal range. These findings indicate that rather than relying on a single time-point measurement, sequential assessment from the preoperative period through the early postoperative phase (up to postoperative day 2) may be important for detecting redox alterations associated with POD.

Practical bedside assays, such as d-ROMs and BAP assays, may potentially support early risk stratification and inform targeted preventive strategies, in the future, pending further validation. However, as this was an observational exploratory study, these results should be interpreted cautiously, and prospective studies are essential to establish their validity as clinical indicators. For clinical implementation, future work should include external validation of predictive performance (e.g., determination of cutoff values, evaluation of AUC and reclassification improvement) and assessment of the feasibility of measurement protocols.

### 4.4. Limitations

This study has some limitations. First, this single-center retrospective observational study had a relatively small sample size, which might have limited the statistical power. Furthermore, adjustments were made for age, operative time, and hypertension status only, and additional unmeasured confounders may exist. Given the multifactorial nature of POD, including inflammatory responses, neuronal injury, and anesthetic depth [46,47,48], the incorporation of a broader range of biomarkers and clinical variables is essential. In addition, only 50 of the 175 eligible patients were included in the final analysis because continuous residual serum samples up to postoperative day 4 could not be technically secured in many cases. In our institution, perioperative blood sampling schedules are routinely standardized for cardiac surgery patients, suggesting that missing specimens were unlikely to be related to clinical severity or ICU course, but rather to limitations in residual sample volume and storage duration. However, including only patients with complete stored specimens may have introduced selection bias, and caution is warranted when generalizing these findings.

Second, POD diagnosis may be constrained by the use of the ICDSC alone. The ICDSC is widely used in routine practice and allows for 24 h monitoring; however, its specificity is lower than that of the Confusion Assessment Method for the ICU when using a cutoff of ≥3 [49], and subjective elements may reduce diagnostic reproducibility. Additionally, limiting POD assessment to postoperative day 4 might have resulted in missed cases of late-onset POD.

Third, despite recognizing preoperative cognitive impairment as a well-known risk factor for POD [2,43], systematic cognitive screening (e.g., Mini-Mental State Examination) was not performed for all patients, potentially contributing to residual confounding.

Finally, multiple cardiac procedures (e.g., valvular, aortic, and CABG surgeries) were included, which may differ in oxidative stress responses and POD incidence [50,51,52,53]. Minimally invasive cardiac surgery, now increasingly common, may elicit distinct inflammatory and redox responses [54]. Although stratified analyses according to surgical procedure were not performed due to sample size limitations, the heterogeneity of surgical types should be considered when interpreting the results, as procedures may differ in oxidative stress response. In addition, this study was conducted at a single institution in a Japanese cohort, and patient backgrounds, surgical techniques, and perioperative management strategies may vary across regions or institutions, limiting the external validity of our findings.

Taken together, to more accurately evaluate the relative contribution of oxidative stress and translate these findings into clinical application, future research should incorporate multivariable models accounting for multiple factors, perform procedure-specific analyses, and include external validation in multicenter cohorts, ideally through large-scale prospective studies incorporating additional biomarkers.

## 5. Conclusions

POD was associated with an unfavorable redox profile characterized by increased preoperative d-ROMs levels, reduced baseline BAP/d-ROMs ratio, transient postoperative increase in BAP values, and relatively higher BAP/d-ROMs ratio in patients who underwent cardiac surgery. Preoperative d-ROMs levels were positively correlated with POD severity. These findings suggest that redox dynamics from the preoperative period to the early postoperative period may play a role in POD development and severity. However, this study was a retrospective observational study conducted in a single-center Japanese cohort, and therefore the generalizability of the findings is limited. Future prospective multicenter studies and external validation using multimodal predictive models are warranted to further evaluate the clinical applicability and feasibility of these redox biomarkers in perioperative monitoring.

## Figures and Tables

**Table 1:** Baseline characteristics and intraoperative variables according to POD status.

	POD (*n* = 18)	No POD (*n* = 32)	*p*-Value
**Demographics**			
Age (years)	75.6 ± 6.7	69.3 ± 11.6	**0.019**
Sex (Male), *n* (%)	10 (55.5%)	18 (56.5%)	1.000
BMI (kg/m^2^)	21.2 ± 3.9	22.4 ± 4.1	0.312
Smoking(ever), *n* (%)	4 (22.2%)	15 (46.8%)	0.130
Daily drinking, *n* (%)	2 (11.1%)	5 (18.8%)	1.000
**Baseline oxidative stress markers**			
pre d-ROMs (U.CARR)	352.4 ± 80.6	312.1 ± 67.6	0.082
pre BAP (µmol/L)	2438.8 ± 234.4	2389.7 ± 254.0	0.495
pre BAP/d-ROMs	7.3 ± 1.7	8.0 ± 1.9	0.188
**Medical history**			
Diabetes, *n* (%)	4 (22.2%)	4 (12.5%)	0.436
Hypertension, *n* (%)	13 (72.2%)	12 (37.5%)	**0.038**
Chronic kidney disease, *n* (%)	3 (16.7%)	6 (18.8%)	1.000
Dyslipidemia, *n* (%)	7 (38.9%)	7 (21.9%)	0.325
Cerebrovascular disease, *n* (%)	4 (22.2%)	4 (12.5%)	0.436
**Procedure characteristics**			
Type of operation, *n* (%)			
CABG	2 (11.1%)	5 (15.6%)	0.157
Aortic Surgery	10 (55.6%)	9 (28.1%)	
Valve Surgery	6 (33.3%)	18 (56.3%)	
Surgical approach, *n* (%)			
MICS	6 (33.3%)	15 (46.9%)	0.388
MS	12 (66.7%)	17 (53.1%)	
Operative time (min)	373 ± 153.3	260.1 ± 74.0	**<0.001**
Anesthesia time (min)	477.9 ± 161.7	353.1 ± 75.6	**<0.001**
Cardiopulmonary bypass time (min)	212.5 ± 84.4	154.2 ± 45.6	**0.002**
Total blood product transfusion (mL)	3692.6 ± 2754.9	2110.8 ± 1620.1	**0.012**

Continuous variables are presented as mean ± standard deviation, whereas categorical variables are expressed as *n* (%). Surgical procedures were categorized into CABG, valve surgery, and aortic surgery and were assigned using a predefined hierarchical rule: any case with aortic intervention was classified as aortic surgery, combined valve and CABG procedures were classified as valve surgery, and the CABG group comprised patients who underwent CABG only. The surgical approach was classified as minimally invasive cardiac surgery versus conventional median sternotomy. Between-group comparisons were performed for descriptive purposes to characterize baseline differences between the POD and non-POD groups. *p*-values were calculated using Student’s *t*-test for continuous variables and Fisher’s exact test for categorical variables. Bold numbers indicate statistically significant differences. Abbreviations: POD: Postoperative delirium; CABG: coronary artery bypass grafting; MICS: Minimally Invasive Cardiac Surgery; MS: Mitral Surgery.

## Data Availability

Data is contained within the article or Supplementary Material.

## Supplementary Materials

The following supporting information can be downloaded at: https://www.mdpi.com/article/10.3390/antiox15010108/s1, Table S1: Reference ranges for derivatives of reactive oxygen metabolites (d-ROMs) and biological antioxidant potential (BAP) assays; Figure S1: Forest plots showing the adjusted mean differences in derivatives of reactive oxygen metabolites (d-ROMs) (A), biological antioxidant potential (BAP) (B), and BAP/d-ROMs ratio (C) between the postoperative delirium (POD) and non-POD groups at each perioperative time point (preoperative, immediately after surgery, postoperative days 1–4); Figure S2: Forest plots showing the adjusted mean differences in the relative change ratios of derivatives of reactive oxygen metabolites (d-ROMs) (A), biological antioxidant potential (BAP) (B), and BAP/d-ROMs ratio (C) between the postoperative delirium (POD) and non-POD groups at each perioperative time point (immediately after surgery, postoperative days 1–4); Figure S3: Forest plots showing the adjusted mean differences in derivatives of reactive oxygen metabolites (d-ROMs) (A), biological antioxidant potential (BAP) (B), and BAP/d-ROMs ratio (C) between the postoperative delirium (POD) and non-POD groups at each perioperative time point (preoperative, immediately after surgery, postoperative days 1–4); Figure S4: Forest plots showing the adjusted mean differences in the relative change ratios of derivatives of reactive oxygen metabolites (d-ROMs) (A), biological antioxidant potential (BAP) (B), and BAP/d-ROMs ratio (C) between the postoperative delirium (POD) and non-POD groups at each perioperative time point (immediately after surgery, postoperative days 1–4); Figure S5: Subgroup analysis according to age (<75 years vs. ≥75 years); Figure S6: Subgroup analysis according to the operative time (<268 min vs. ≥268 min); Figure S7: Subgroup analysis according to hypertension status (presence vs. absence).

## Abbreviations

The following abbreviations are used in this manuscript:

BAP	Biological antioxidant potential
CABG	Coronary artery bypass grafting
d-ROMs	Derivatives of reactive oxygen metabolites
ICDSC	Intensive Care Delirium Screening Checklist
LS	Least-squares
MMRM	Mixed-effects model for repeated measures
POD	Postoperative delirium
RASS	Richmond Agitation–Sedation Scale
SE	Standard error
