# Supplementary material for: Temporal Dynamics of Perioperative Redox Balance and Its Association with Postoperative Delirium After Cardiac Surgery
Source: Antioxidants (Basel). 2026 Jan 14;15(1):108. doi: 10.3390/antiox15010108 (PMC12837909; doi:10.3390/antiox15010108)
Supplement: Supplementary file 1 [file antioxidants-15-00108-s001.zip › antioxidants-4060751-supplementary.pdf]

Supplementary Materials

Table S1. Reference ranges for derivatives of reactive oxygen metabolites (d-ROMs) and biological antioxidant potential (BAP) assays.

**d-ROMs Test**	(U.CARR)
Normal values	200-300
Borderline values	301-320
Low-level oxidative stress	321-340
Middle-level oxidative stress	341-400
High-level oxidative stress	401-500
Very high-level oxidative stress	501-

**BAP Test**	(μmol/L)
Normal values	2200-
Borderline values	2000-2200
Slight reduction	1800-2000
Moderate reduction	1600-1800
Strong reduction	1400-1600
Very strong reduction	-1400

d-ROMs values are expressed in Carratelli units (U. CARR), where 1 U. CARR corresponds to 0.08 mg/100 mL of hydrogen peroxide equivalents. BAP values are expressed in μmol/L. These reference ranges are based on the manufacturer's standard values.

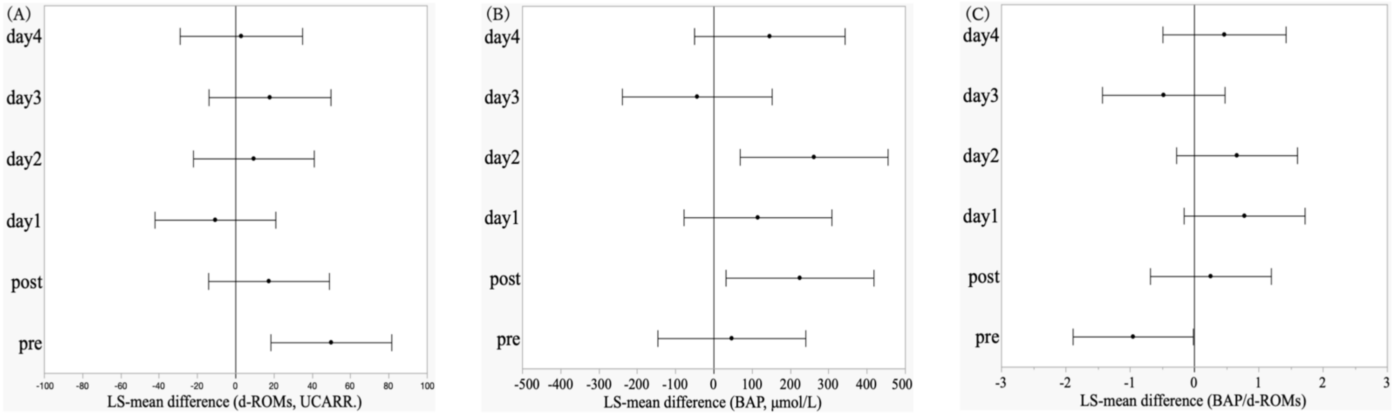

Figure S1. Forest plots showing the adjusted mean differences in derivatives of reactive oxygen metabolites (d-ROMs) (**A**), biological antioxidant potential (BAP) (**B**), and BAP/d-ROMs ratio (**C**) between the postoperative delirium (POD) and non-POD groups at each perioperative time point (preoperative, immediately after surgery, postoperative days 1–4). Mean differences (POD minus non-POD) and 95% confidence intervals were estimated using a mixed-effects model for repeated measures (MMRM)**.** Positive values indicate higher levels in the POD group than in the non-POD group.

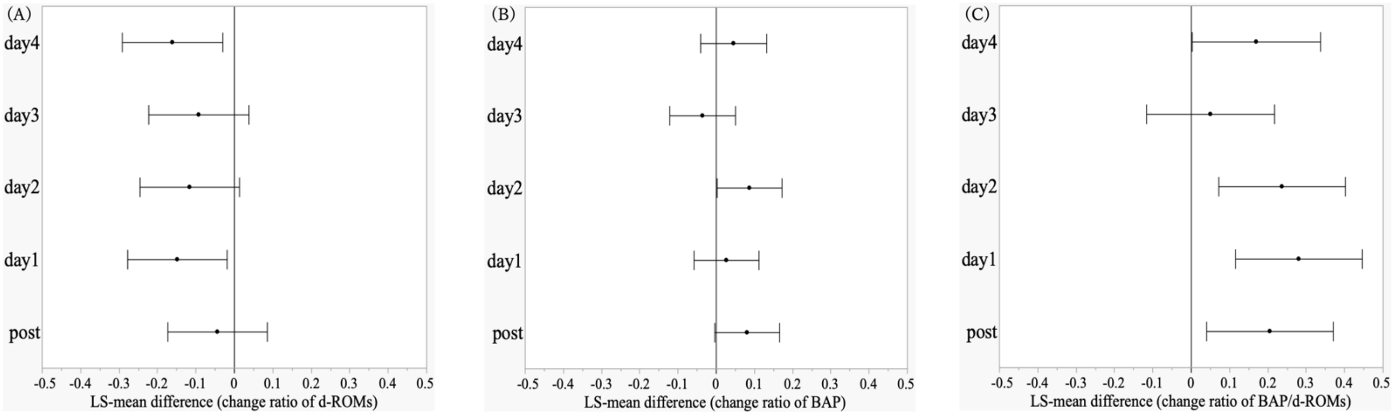

Figure S2. Forest plots showing the adjusted mean differences in the relative change ratios of derivatives of reactive oxygen metabolites (d-ROMs) (**A**), biological antioxidant potential (BAP) (**B**), and BAP/d-ROMs ratio (**C**) between the postoperative delirium (POD) and non-POD groups at each perioperative time point (immediately after surgery, postoperative days 1–4). The relative change ratio was calculated as the value at each time point divided by the preoperative baseline value (set as 1.0). Mean differences (POD minus non-POD) and 95% confidence intervals were estimated using a mixed-effects model for repeated measures (MMRM). Positive values indicate greater relative increases in the POD group than in the non-POD group.

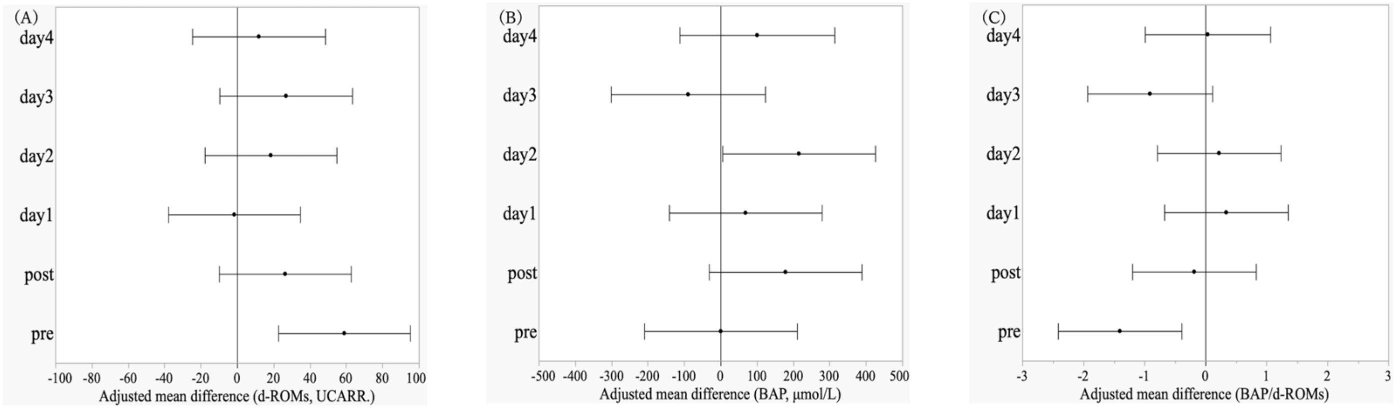

Figure S3. Forest plots showing the adjusted mean differences in derivatives of reactive oxygen metabolites (d-ROMs) (**A**), biological antioxidant potential (BAP) (**B**), and BAP/d-ROMs ratio (**C**) between the postoperative delirium (POD) and non-POD groups at each perioperative time point (preoperative, immediately after surgery, postoperative days 1–4). Adjusted mean differences (POD minus non-POD) and 95% confidence intervals were estimated using a mixed-effects model for repeated measures (MMRM), with age, operative time, and hypertension as the covariates. Horizontal bars represent 95% confidence intervals. Positive values indicate higher levels in the POD group than in the non-POD group after adjusting for covariates.

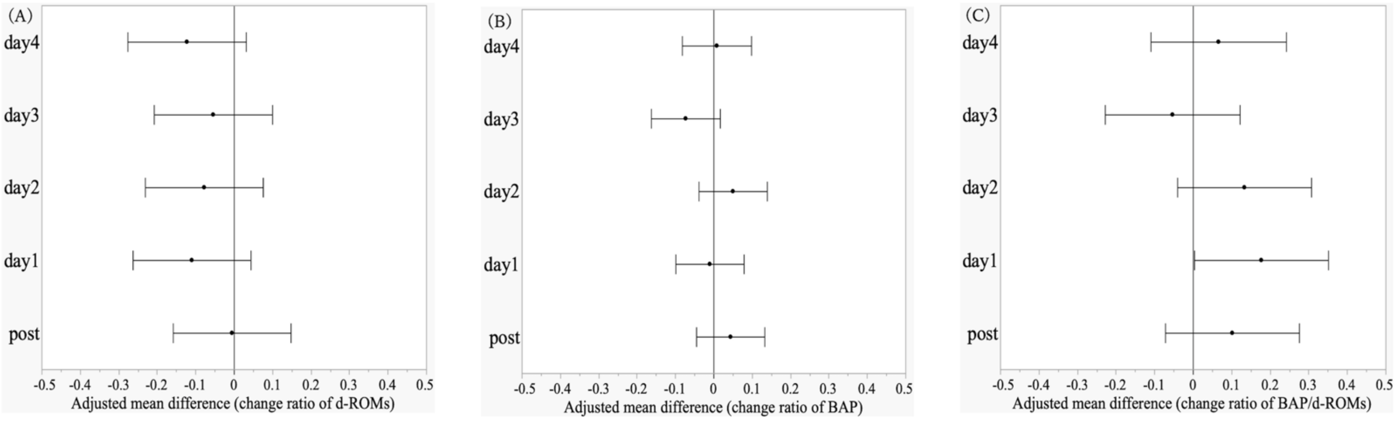

Figure S4. Forest plots showing the adjusted mean differences in the relative change ratios of derivatives of reactive oxygen metabolites (d-ROMs) (**A**), biological antioxidant potential (BAP) (**B**), and BAP/d-ROMs ratio (**C**) between the postoperative delirium (POD) and non-POD groups at each perioperative time point (immediately after surgery, postoperative days 1–4). The relative change ratio was calculated as the value at each time point divided by the preoperative baseline value (set as 1.0). Mean differences (POD minus non-POD) and 95% confidence intervals were estimated using a mixed-effects model for repeated measures (MMRM), with age, operative time, and hypertension as the covariates. Positive values indicate greater relative increases in the POD group than in the non-POD group.

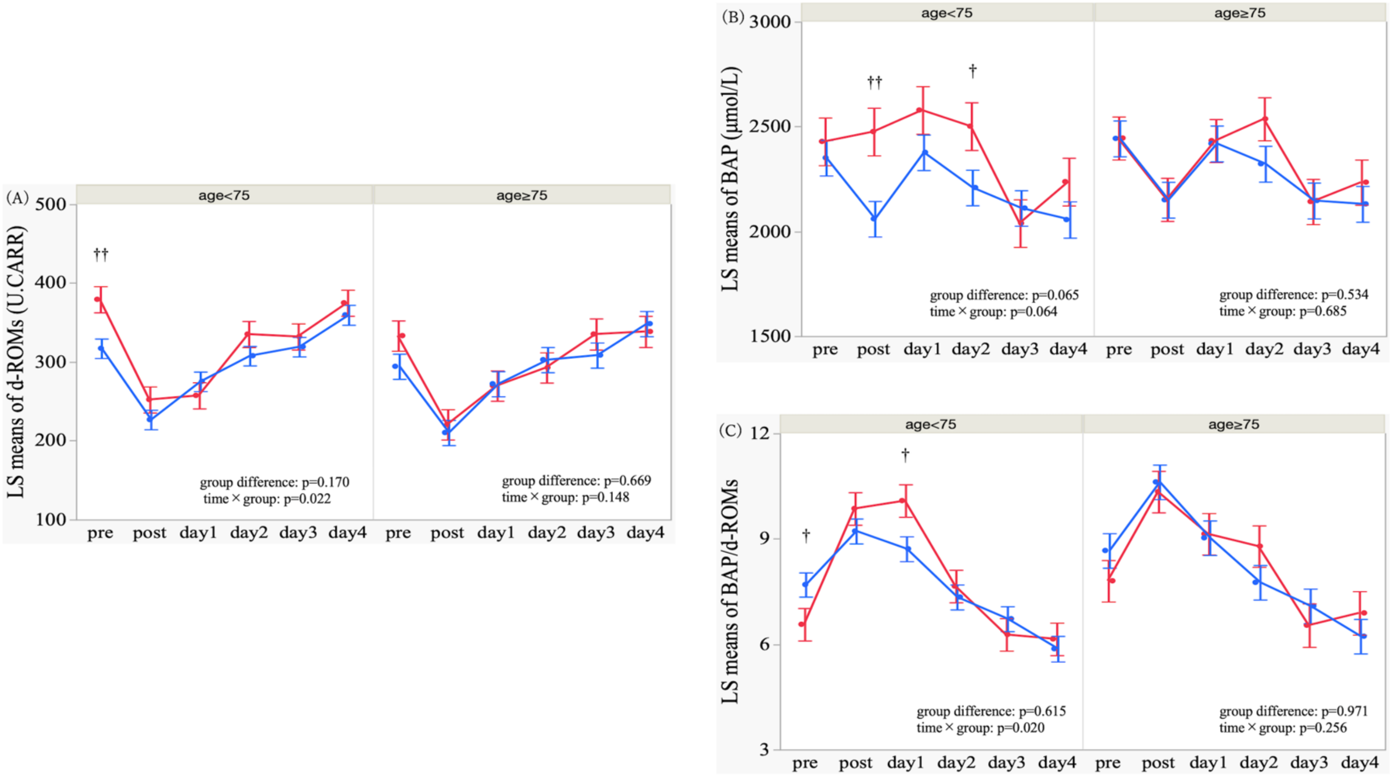

Figure S5. Subgroup analysis according to age (<75 years vs. ≥75 years). Longitudinal profiles of (**A**) derivatives of reactive oxygen metabolites (d-ROMs), (**B**) biological antioxidant potential (BAP), and (**C**) BAP/d-ROMs ratio in patients with and without postoperative delirium (POD), stratified by age group. At each time point, values are presented as least-squares (LS) means estimated using a mixed-effects model for repeated measures (MMRM). Lines connect the LS means across time, and error bars indicate the standard error (SE). The time points were as follows: pre = preoperative baseline; post (day 0) = immediately after surgery at ICU admission; days 1–4 = postoperative days 1–4. Red indicates POD (+), whereas blue denotes POD (−). The p-values shown in each panel correspond to tests for the group differences (POD vs non-POD) and the time × group interaction (fixed effects) in the MMRM. †: $p < 0.05$, ††: $p < 0.01$ for between-group comparisons at each time point. Age groups were dichotomized at 75 years (an age of 75 years was chosen as the cutoff because it is commonly used to define elderly patients in clinical and epidemiological studies). The number of POD (+) versus POD (−) patients was 10 versus 18 for <75 years and 9 versus 13 for ≥75 years, respectively.

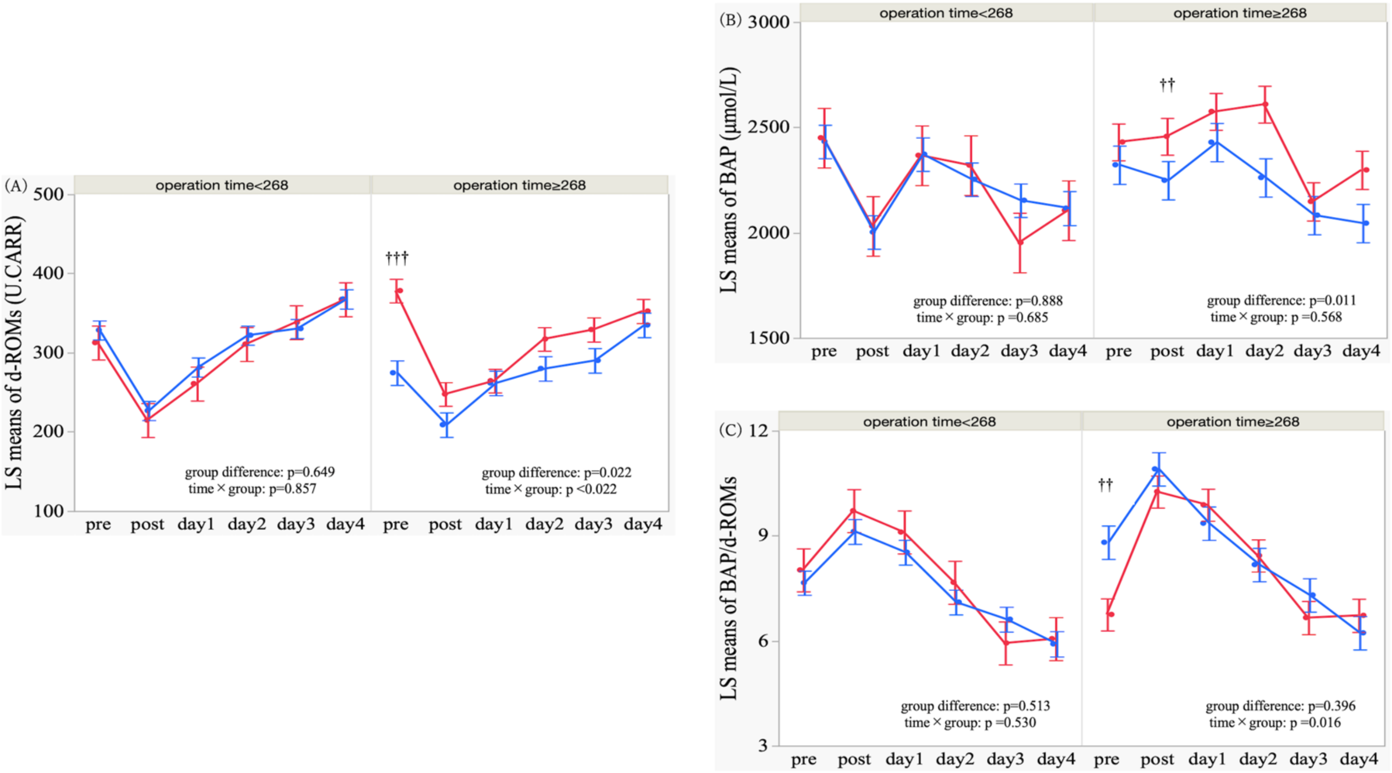

Figure S6. Subgroup analysis according to the operative time (<268 min vs. ≥268 min). Longitudinal profiles of (**A**) derivatives of reactive oxygen metabolites (d-ROMs), (**B**) biological antioxidant potential (BAP), and (**C**) BAP/d-ROMs ratio in patients with and without postoperative delirium (POD), stratified by the operative time. At each time point, values are presented as least-squares (LS) means estimated using the mixed-effects model for repeated measures (MMRM). Lines connect the LS means across time, and error bars represent the SE. The time points were as follows: pre = preoperative baseline; post (day 0) = immediately after surgery at ICU admission; days 1–4 = postoperative days 1–4. Red indicates POD (+), whereas blue denotes POD (−). The p-values shown in each panel correspond to tests for the group differences (POD vs non-POD) and the time × group interaction (fixed effects) in the MMRM. †: $p < 0.05$, ††: $p < 0.01$, †††: $p < 0.001$ for between-group differences at each time point. Patients were divided using a median operative time of 268 min. The number of POD (+) versus POD (−) patients was 6 versus 19 for <268 min and 13 versus 12 for ≥268 min, respectively.

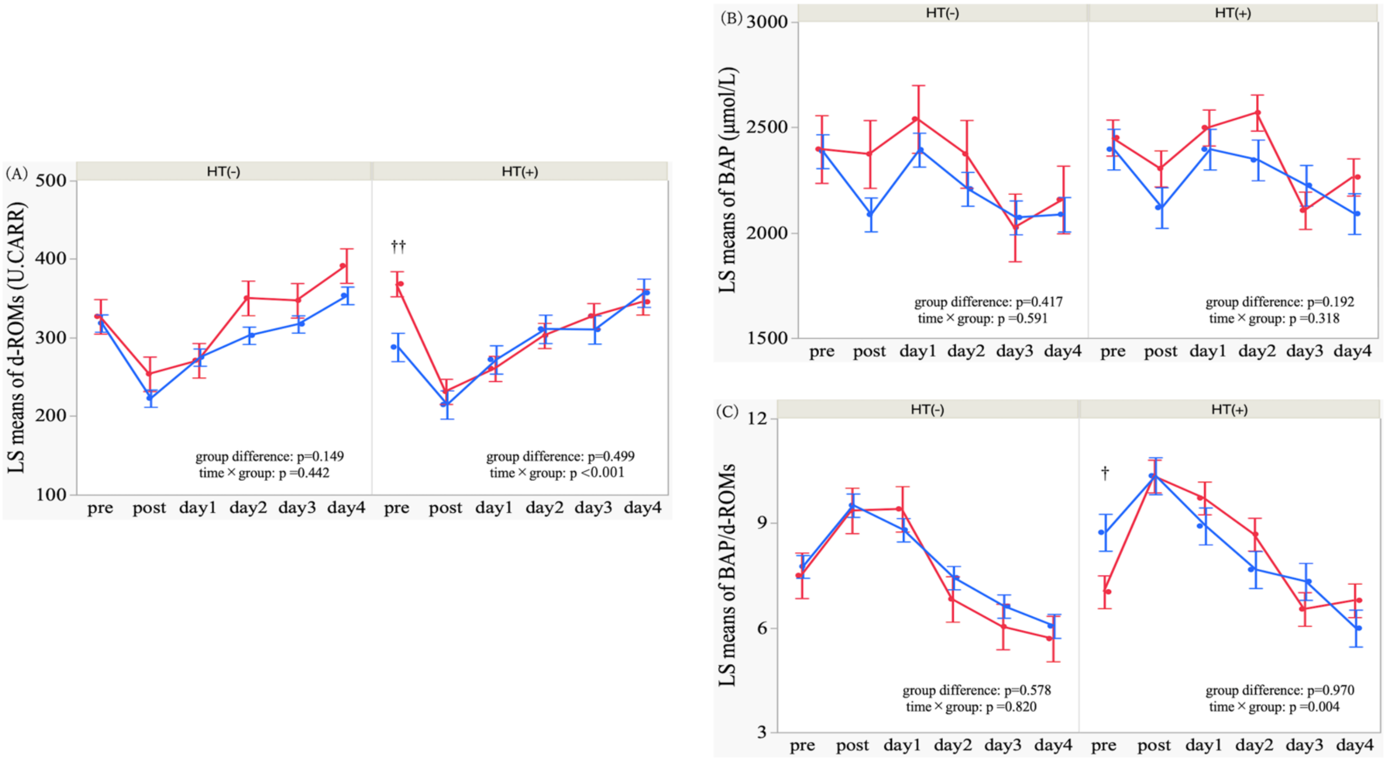

Figure S7. Subgroup analysis according to hypertension status (presence vs. absence). Longitudinal profiles of (**A**) derivatives of reactive oxygen metabolites (d-ROMs), (**B**) biological antioxidant potential (BAP), and (**C**) BAP/d-ROMs ratio in patients with and without postoperative delirium (POD), stratified by hypertension status. At each time point, values are presented as least-squares (LS) means estimated using the mixed-effects model for repeated measures (MMRM). Lines connect the LS means across time, and error bars represent the SE. The time points were as follows: pre = preoperative baseline; post (day 0) = immediately after surgery at ICU admission; days 1–4 = postoperative days 1–4. Red indicates POD (+), whereas blue denotes POD (−). The p-values shown in each panel correspond to tests for the group differences (POD vs non-POD) and the time × group interaction (fixed effects) in the MMRM. †: $p < 0.05$, ††: $p < 0.01$ for between-group comparisons at each time point. The number of POD (+) versus POD (−) patients was 5 versus 20 for the absence of hypertension and 13 versus 12 for the presence of hypertension, respectively.
